# Optimizing intervention components for sleep promotion in children in the context of obesity prevention: the SLEEPY 2.0 study protocol

**DOI:** 10.3389/frsle.2023.1264532

**Published:** 2023-10-04

**Authors:** Maddy Fair, Jessica Decker, Alexander G. Fiks, Stephanie Mayne, Knashawn H. Morales, Ariel A. Williamson, Jonathan A. Mitchell

**Affiliations:** ^1^Division of Gastroenterology, Hepatology, and Nutrition, Children's Hospital of Philadelphia, Philadelphia, PA, United States; ^2^Department of Pediatrics, Perelman School of Medicine, University of Pennsylvania, Philadelphia, PA, United States; ^3^Department of Biostatistics, Epidemiology and Informatics, Perelman School of Medicine, University of Pennsylvania, Philadelphia, PA, United States; ^4^Department of Child and Adolescent Psychiatry and Behavioral Sciences, Children's Hospital of Philadelphia, Philadelphia, PA, United States

**Keywords:** insufficient sleep, obesity, children, sleep, pediatrics, intervention

## Abstract

**Background:**

Insufficient sleep duration is highly prevalent in childhood and is associated with obesity, especially among middle school-aged children. The primary care setting has enormous potential to promote sleep, but limited time and sleep resources at in person appointments are key barriers. Digital health innovations offer solutions to these barriers. Mobile health platforms can be developed to deliver behavioral sleep promotion remotely in the home setting, with tailoring to individual and contextual factors to help ensure equitable effectiveness across sociodemographic groups. This paper presents the protocol for a randomized optimization trial using the Multiphase Optimization Strategy (MOST) to develop a mobile health platform for the pediatric care setting to promote longer sleep duration for childhood obesity prevention.

**Methods:**

This is a single-site study being conducted at the Children's Hospital of Philadelphia. We will randomize 325 children, aged 8–12 y, with a body mass index (BMI) between the 50th−95th percentile, and who sleep <8.5 h per night. The Way to Health mobile platform will facilitate remote communication and data collection. A sleep tracker will estimate sleep patterns for 12-months (2-week run-in; 6-month intervention; ≈5.5-month follow-up). A randomized 2^4^ factorial design will assess four components: sleep goal (≥9 h or ≥30 min above baseline sleep duration), digital guidance (active or active with virtual study visits), caregiver incentive (inactive or active), and performance feedback (inactive or active). Fat mass will be measured at baseline, 6-, and 12-months using dual energy X-ray absorptiometry. Total energy intake and the timing and composition of meals will be measured using 24-h dietary recalls at baseline, 6-, and 12-months. Sociodemographic data (e.g., sex, race, ethnicity) will be measured using self-report and home addresses will be geocoded for geospatial analyses.

**Discussion:**

We anticipate that this innovative optimization trial will identify optimal component settings for sleep promotion in children, with clinically meaningful improvements in fat mass trajectories. Importantly, the platform will have broad impact by promoting sleep health equity across sociodemographic groups. With the optimal settings identified, we will be able to determine the effectiveness of the final intervention package under the evaluation phase of the MOST framework in a future randomized controlled trial. Our proposed research will greatly advance the field of behavioral sleep medicine and reimagine how insufficient sleep duration and obesity are prevented in pediatric healthcare.

**Trial registration:**

ClinicalTrials.gov NCT05703347 registered on 30 January 2023.

## 1. Introduction

### 1.1. Childhood obesity

In the 1960s the prevalence of obesity among U.S. children was 5% (Troiano et al., 1995), and it increased three-fold to 15% by the late 1990s (Ogden et al., 2002). The prevalence increased further to 18% by the late 2000s (Ogden et al., 2010, 2012) and has remained at this excessively high level into the present (Ogden et al., 2014). Childhood obesity affects both sexes equally but is disproportionally higher among Black children and children from lower socioeconomic backgrounds (Ogden et al., 2018). Childhood obesity is associated with multiple co-morbidities and chronic diseases in later life, especially cardiovascular diseases (Bjorge et al., 2008; Tirosh et al., 2011; Steinberger et al., 2016; Twig et al., 2016). Further, childhood obesity tracks into adulthood (The et al., 2010; Rundle et al., 2020), and if left unchecked an estimated 57% of children born today will be obese when they are 35 years old (Ward et al., 2017). A key Healthy People 2030 goal is to reduce the prevalence of childhood obesity to 15.5% (Healthy People 2030, 2021b). Meeting this goal will require a multi-sector approach and the pediatric healthcare setting has a critical role. Indeed, an American Academy of Pediatrics Clinical Report detailed the pediatrician's role in preventing childhood obesity and highlighted sleep promotion as a preventive measure that is needed in pediatric healthcare (Daniels et al., 2015).

### 1.2. Sleep and pediatric obesity

In 2008, two independent meta-analyses of cross-sectional studies reported that shorter sleep duration was associated with childhood obesity as defined by body mass index (BMI) (Cappuccio et al., 2008; Chen et al., 2008). The meta-analysis by Chen et al. included 10 cross-sectional studies and reported that shorter sleep duration was associated with a 1.58 (95% CI: 1.26–1.98) increased odds of childhood obesity (Chen et al., 2008). The meta-analysis by Cappuccio et al. included 11 cross-sectional studies and reported that shorter sleep duration was associated with a 1.89 (95% CI: 1.46–2.43) increased odds of obesity (Cappuccio et al., 2008). Meta-analyses involving longitudinal studies have been completed and provide evidence of temporality, with shorter sleep duration leading to childhood obesity (Fatima et al., 2015; Ruan et al., 2015; Miller et al., 2018). Indeed, the latest meta-analysis of longitudinal studies reported that shorter sleep duration was associated with the incidence of overweight and obesity across all ages in childhood, with the strongest association observed in middle school aged children (RR = 2.23, 95%CI: 2.21–2.27) (Miller et al., 2018). Shorter sleep has been associated with other obesity phenotypes in childhood, including waist circumference and estimates of fat mass using bioelectrical impedance, dual energy X-ray absorptiometry, and skinfold thickness techniques (Garaulet et al., 2011; Cespedes et al., 2014; Martinez et al., 2014; Mcneil et al., 2015; Collings et al., 2017; Derks et al., 2017; Nam et al., 2017; Cespedes Feliciano et al., 2018, 2019).

### 1.3. Sleep and obesity: biological plausibility

It has been proposed that shorter sleep leads to a positive energy balance by increasing energy (calorie) intake and/or decreasing physical activity energy expenditure (Taheri, 2006). For the latter, the logic is that shorter sleep leads to tiredness and fatigue and therefore less physical activity, but evidence for this mechanism is mixed (Ortega et al., 2010, 2011; Foti et al., 2011; Mitchell et al., 2016; Hart et al., 2017; Vincent et al., 2017; Armstrong et al., 2021; Jindal et al., 2021). In contrast, there is compelling evidence that shorter sleep duration is associated with increased energy intake (Weiss et al., 2010; St-Onge et al., 2011; Markwald et al., 2013; Spaeth et al., 2013; Fisher et al., 2014; Hjorth et al., 2014; Kjeldsen et al., 2014; Franckle et al., 2015; Hunsberger et al., 2015; Al Khatib et al., 2017; Martinez et al., 2017; Rangan et al., 2018; Mi et al., 2019). The simplest explanation is that more time awake allows for more opportunities to eat. However, more complex biology may be involved. First, the majority of the additional calories are consumed in the late evening (Markwald et al., 2013; Spaeth et al., 2013). Night eating may be particularly obesogenic and restricting food intake to be aligned with circadian rhythms could help to prevent obesity (Arble et al., 2009; Panda, 2016; Xiao et al., 2019; Chow et al., 2020; Phillips et al., 2021). For instance, mice fed a high fat diet gained excessive weight only when they were free to eat throughout their 24-h sleep-wake cycle and not when food access was restricted to the dark period (equivalent of daytime in nocturnal humans) (Hatori et al., 2012); and there are emerging data that time restricted feeding improves weight and cardiometabolic health in humans (Chow et al., 2020; Wilkinson et al., 2020; Phillips et al., 2021). Second, experimental sleep restriction in adults has been shown to cause changes in hormones that regulate energy homeostasis in the hypothalamus that could promote increased energy intake (e.g., reductions in leptin and increases in ghrelin) (Spiegel et al., 2004a,b); however, this hormonal hypothesis is not universally supported (Hitze et al., 2009; Nedeltcheva et al., 2009; St-Onge et al., 2011; Hart et al., 2013; Markwald et al., 2013; Boeke et al., 2014). Third, shorter sleep may alter how the brain processes food cues, increasing temptation to eat (St-Onge et al., 2012, 2014; Demos et al., 2017). Functional magnetic resonance imaging has revealed that brain regions associated with reward are more active upon viewing unhealthy foods after sleep restriction, compared to healthy sleep conditions (St-Onge et al., 2014). It has also been proposed that shorter sleep can up-regulate the odor-processing region of the brain and this heightened sense of smell may lead to increased consumption energy-dense foods (Bhutani et al., 2019). Finally, sleep-restriction impairs cognition, which may affect rational decision-making, including decisions about foods consumption (Pardi et al., 2017).

### 1.4. Behavioral sleep promotion strategies in childhood

The American Academy of Sleep Medicine recommends that children (6–12y) and adolescents (13–18y) sleep for 9–12 h and 8–10 h per day, respectively (Paruthi et al., 2016). However, data from the Youth Risk Behavior Surveillance Survey revealed that most middle school (53%) and high school students (65%) do not meet these recommendations (Wheaton et al., 2016, 2018). A Healthy People 2030 goal is to reduce the prevalence of insufficient sleep in childhood to 30% (Healthy People 2030, 2021a). Emerging data demonstrate that it is feasible to increase sleep duration among children using behavioral strategies. A meta-analysis revealed that behavioral sleep promotion interventions effectively increase sleep duration by an average of 35 (95% CI: 9, 61) minutes per night, in adolescents and young adults (Griggs et al., 2020). There is not yet a consensus on the specific behavioral components that are needed to promote sleep in children, but common elements include: setting a sleep goal, limiting sleep barriers, and enhancing sleep facilitators (Hart et al., 2016; Perfect et al., 2016; Meltzer et al., 2021a). Further, there is evidence that providing financial incentives can facilitate behavior change (Patel et al., 2016; Wong et al., 2017), and this needs to be considered for child sleep promotion efforts.

#### 1.4.1. Sleep goal

Setting a sleep goal is a key intervention component for sleep extension (Hart et al., 2016; Perfect et al., 2016; Van Dyk et al., 2017), but the best approach to setting a sleep goal is unknown. The American Academy of Sleep Medicine recommends that middle school aged children sleep for 9–12 h each day (Paruthi et al., 2016), so in this age-range a guideline-based goal within this range is logical. However, Self-Efficacy Theory predicts that children will be more motivated to increase their sleep duration if they believe that they can attain the sleep extension goal (Bandura, 1977). Accordingly, a 7-h per night sleeper would likely have lower self-efficacy for a guideline-based goal than an 8-h per night sleeper. A personalized goal approach would minimize any differential self-efficacy arising from baseline sleep duration and may be more effective for childhood sleep promotion.

Providing feedback based on sleep goal achievement and other sleep metrics may be critical. Timely, personalized, and performance feedback on goal achievement may help to increase enjoyment and competence and thereby intrinsic motivation. Further, providing performance feedback to participants and the primary care improved the effectiveness of an obesity treatment intervention in adults (Spring et al., 2020), and this needs to be tested in the context of childhood obesity prevention.

#### 1.4.2. Barriers and facilitators

Barriers to optimal sleep duration include evening electronics usage, caffeine intake, excessive extracurricular schedules, and a lack of parental oversight regarding consistent bedtimes. Electronic screens emit blue wavelength light and when exposure is high in the evening it inhibits melatonin secretion that is needed to induce sleep (Lee et al., 2018). Television viewing is common in children, but with the increasing availability of streaming services and social network platforms, a large proportion of youth now use computers, tablets, and smartphones (Li et al., 2010; Ogunleye et al., 2015; Yland et al., 2015; Brunetti et al., 2016; Council on Communications Media, 2016; Dube et al., 2017; Twenge et al., 2017). Children and adolescents spend in excess of 7 h per day engaged in electronic media (Rideout et al., 2010). Not related to school work, 43% of children in the U.S. spend ≥3 h per day using a computer (including for texting and using social media) (Kann et al., 2018). Experimental studies suggest that targeting reductions in screen time can help youth extend their sleep in the home setting (Perfect et al., 2016; Hart et al., 2017; Van Dyk et al., 2018; Perrault et al., 2019). Indeed, exit survey data from one experimental study revealed that removing electronics from bedrooms and recording TV shows were useful strategies to extend child sleep (Perfect et al., 2016). In a qualitative study, youth reported being aware that excessive technology use is a sleep barrier, and were open to curbing use to improve sleep (Paterson et al., 2019).

Caffeine is a stimulant that promotes wakefulness and has been associated with insufficient sleep (Pollak and Bright, 2003; Calamaro et al., 2009, 2012; Li et al., 2010; Franckle et al., 2015). Children are encouraged to limit their caffeine intake to no more than 100 mg per day (Committee on Nutrition the Council on Sports Medicine Fitness, 2011), and to especially limit caffeine intake in the late afternoon and evening (Bryant Ludden and Wolfson, 2010). In the U.S., 75% of children consume caffeine daily (Branum et al., 2014). Caffeine is often a “hidden” ingredient in food products; for example, sports drinks are often caffeinated and regularly consumed by children who play on after-school sports teams (Committee on Nutrition the Council on Sports Medicine Fitness, 2011; Bernstein et al., 2018). Reducing/removing this stimulant from diets would help address insufficient sleep (Buxton et al., 2015).

Children are more likely to go to bed earlier and sleep sufficiently if their parents set consistent bedtimes (Li et al., 2010; Short et al., 2011; Appelhans et al., 2014; Buxton et al., 2015; Pyper et al., 2017). However, only 6% of parents in the U.S. have bedtime rules for their child (Short et al., 2011, 2013). In a sleep extension study, families reported that doing homework earlier, setting bedtimes and planning ahead were useful strategies to help increase sleep duration (Perfect et al., 2016). Children should gain autonomy as they mature, but parents should remain committed to setting consistent bedtimes (Haines et al., 2016).

Youth in the U.S. spend more than 2.5 h per night engaged in extracurricular activities that can delay bedtimes and reduce sleep duration (Short et al., 2013). In contrast, youth in Australia spend 1.4 h per night engaged in extracurricular activities (Short et al., 2013). Planning ahead and adjusting these activities could benefit child sleep duration (Haines et al., 2016).

#### 1.4.3. Incentives

Despite best intentions, individuals are often non-adherent to prescribed medications, treatments, and preventive guidelines (McNally et al., 2009; Asch et al., 2012; Yoon et al., 2015; Rotenberg et al., 2016; Xanthopoulos et al., 2017; Hall et al., 2018; Biederman et al., 2019), including for childhood obesity prevention (Frank et al., 2020). This is predictable and behavioral economists have devised strategies, or nudges, to counteract such behavior (Frank et al., 2020), including the delivery of financial incentives to motivate behavior change (Loewenstein et al., 2012; Thirumurthy et al., 2019). The strategy used to deliver financial incentives can have a large impact on effectiveness. For example, loss-framed incentives have been shown to be more effective than gain-framed incentives, of equal value, to help promote physical activity levels in adults (Patel et al., 2016). Further, a loss-framed incentive increased glucose monitoring in adolescents with type I diabetes at a rate more than two times higher than the control arm (Wong et al., 2017). Under gain-framed incentive structures, financial rewards are accumulated if behavior change goals are achieved. Whereas loss-framed incentive structures provide an upfront endowment with financial deductions applied if behavior change goals are not achieved. Based on Prospect Theory, the loss-framed structure is more motivational because we are loss averse (Tversky and Kahneman, 1981). The incentive should also be delivered promptly to provide immediate gratification (Thirumurthy et al., 2019) and has the potential to have lasting behavioral effects through habit formation (Loewenstein et al., 2016). A number of states have received waivers to enact Healthy Behavior Incentive Programs (Huf et al., 2018), and private insurers are increasingly offering enrollees discounted premiums if behavioral targets, measured by wearable devices, are achieved (Raber et al., 2019). These data indicate that incentives for adhering to behavior goals can be implemented into our healthcare system if they are effective.

### 1.5. Sociodemographic context

In the U.S., most children do not meet the recommended sleep duration guidelines, but the extent of this problem is not equally distributed across sociodemographic groups (Guglielmo et al., 2018; Jackson et al., 2020). The state of Pennsylvania, and the city of Philadelphia, are among the lowest ranked in the nation with 79% and 83% of high school students sleeping insufficiently (Wheaton et al., 2018). Black children sleep less than White children (Biggs et al., 2013; Matthews et al., 2014; Guglielmo et al., 2018; Hoyt et al., 2018; Yip et al., 2020), and Hispanic/Latinx children sleep less than non-Hispanic/Latinx children (Combs et al., 2016). There is some evidence that male children sleep less than female children (Moore et al., 2011; Matthews et al., 2014; Mitchell et al., 2020), but other studies report shorter sleep in female children (Hysing et al., 2013; Keyes et al., 2015; Lin et al., 2018; Kocevska et al., 2021). In addition, sleep duration is typically shorter among children living in households with lower incomes (Biggs et al., 2013) and lower parent education levels (Marco et al., 2011; Komrij et al., 2021). The reasons for these disparities in sleep duration are multi-factorial and may be explained in part by family and neighborhood factors (Billings et al., 2021). Parental support will be needed if children are to successfully increase their sleep duration using behavioral strategies (Spilsbury et al., 2005; Heerman et al., 2017; Tsai et al., 2018; Martinez et al., 2019); this may be more challenging in single-parent households (Troxel et al., 2014) and in the context of poor parent-child relationships (Rojo-Wissar et al., 2020). There is also evidence that sleep duration is shorter in neighborhoods with lower socioeconomic status (El-Sheikh et al., 2013; Troxel et al., 2017; Bagley et al., 2018; Sheehan et al., 2018), higher crime (Bailey et al., 2005; Bagley et al., 2016; Heissel et al., 2018; Philbrook et al., 2020), lower social cohesion (Pabayo et al., 2014; Hawkins and Takeuchi, 2016; Troxel et al., 2017; Williamson et al., 2019), higher noise levels (Tiesler et al., 2013; Skrzypek et al., 2017; Weyde et al., 2017), lower tree canopy cover (Mayne et al., 2021), and lower air quality (Husain et al., 2023). These neighborhood factors may impact the success of sleep promotion strategies. These data indicate that sleep interventions should be tailored to individual and contextual factors and evaluated for effectiveness across sociodemographics to address sleep health equity (Guglielmo et al., 2018; Jackson et al., 2020).

### 1.6. Digital solutions for sleep promotion in the pediatric healthcare setting

Pediatric healthcare clinicians adopt a prevention- and family-centered care model (Alley et al., 2019). The vast majority of families seek age-appropriate preventive care at annual well visits (Uddin et al., 2016). Pediatricians endorse the importance of assessing sleep habits and providing sleep-related guidance to families at well visits (Faruqui et al., 2011; Owens and Jones, 2011). However, sleep problem screening and preventive care are severely lacking (Honaker and Meltzer, 2016). Time is a key barrier for pediatric care teams (Honaker and Meltzer, 2016). Digital health solutions could overcome this barrier, with sleep promotion delivered remotely, using mobile health approaches, at regular intervals between annual well visits when sleep is actually taking place in the home setting (Asch et al., 2012). It is increasingly feasible to use wearable devices to track sleep patterns (Meltzer et al., 2015; de Zambotti et al., 2016; Lee et al., 2019), which can be incorporated into mobile health platforms (Asch et al., 2012; Sim, 2019). This allows children to self-monitor sleep patterns and goals. There is accumulating evidence that mobile health systems and telemedicine can deliver effective pediatric healthcare (Singh et al., 2014; Fedele et al., 2017; Zieve et al., 2017; Byrne et al., 2018; Fiks et al., 2018; Foster et al., 2019; Kenyon et al., 2019; Marcus et al., 2019; Novick et al., 2019), especially if parents are actively engaged in the remotely delivered care (Fedele et al., 2017). Furthermore, digital delivery of incentives—designed using behavioral economic theories—has been shown to enhance the effectiveness of mobile health interventions (e.g., physical activity promotion and blood glucose monitoring) (Patel et al., 2016; Wong et al., 2017). Incentives provide a source of immediate gratification directly related to behavior change, which is more motivating than waiting months-to-years for improvements in obesity and related cardiovascular health. Despite this promise, we do not know if digital approaches can be combined to improve sleep duration in childhood for obesity prevention (Allman-Farinelli et al., 2020; Fowler et al., 2021). In fact, a recent systematic review was not able to make any conclusions on mobile health interventions to promote sleep and obesity prevention in children, because the only studies in this area are based on adults (Allman-Farinelli et al., 2020).

### 1.7. Proof of principle: promoting sleep using a mobile health platform

We have successfully deployed a mobile health platform with an integrated sleep tracker, digital messaging, and a behavioral economic inspired incentive for sleep promotion among typically developing children, aged 9–12y, at the Children's Hospital of Philadelphia (Mitchell et al., 2021). The digital messaging components provided feedback on sleep goal achievement and included general sleep health information (i.e., information about bedtime electronics usage) (Mitchell et al., 2021). The behavioral economic component delivered financial incentives to parents based on child sleep goal achievements (Mitchell et al., 2021). We specifically explored the effectiveness of a gain-framed incentive (i.e., accumulated money if sleep goals were achieved) and a loss-framed incentive (i.e., money deducted from an upfront endowment if sleep goals were not achieved) (Mitchell et al., 2021). Based on Prospect Theory, the loss-framed structure should be more motivational because of loss aversion (Tversky and Kahneman, 1981). Interestingly, compared to the control arm, the loss-framed incentive arm increased sleep duration by an average of 34 min per night and increased the probability of sufficient sleep (≥9 h per night) by an average of 18 percentage points (Mitchell et al., 2021). There was no change in sleep duration in those randomized to the gain-framed incentive. Importantly, qualitative data revealed that barriers to sleep extension were primarily related to competing academic and extracurricular demands, as well as sibling activities and caregiver work schedules (Mitchell et al., 2021). In addition, families reported challenges around creating consistent rules related to electronics usage (Mitchell et al., 2021). Facilitators were also noted for future intervention. Children desired more personalized messaging to target their specific sleep habits, while parents suggested enhancing sleep health messaging content (Mitchell et al., 2021).

### 1.8. Optimizing intervention components for sleep promotion

The Multiphase Optimization Strategy (MOST) is an engineer-inspired framework for developing multi-component interventions. MOST has informed a number of multi-component interventions, including: smoking cessation (Piper et al., 2016, 2018; Bernstein et al., 2018; Engle et al., 2019), mental health promotion (Watkins et al., 2016; Uwatoko et al., 2018), physical activity promotion (Celano et al., 2018; Phillips et al., 2018), adult obesity treatment (Pellegrini et al., 2014; Spring et al., 2020), and HIV treatment (Collins et al., 2016; Gwadz et al., 2017). It is ideally suited to determine if digital approaches can be combined and harnessed to improve child sleep duration for the prevention of obesity.

The MOST framework has three phases: (1) the *preparation phase*, to pilot test intervention components; (2) the *optimization phase*, to determine candidate component settings that achieve optimization criteria; and (3) the *evaluation phase*, to determine if the final optimized intervention package is effective using a randomized controlled trial study design (Collins, 2018). With our successful deployment of a mobile health platform to promote sleep in children, we have already completed the preparation phase and are poised to complete the optimization phase (Mitchell et al., 2021). The optimization phase is especially important for developing multi-component interventions as it provides insight into how candidate intervention components operate with respect to primary outcomes. Using highly innovative, efficient and fully powered experimental designs (Collins et al., 2014), optimization trials can determine if each candidate component associates with the primary outcome of interest and can determine if there are interactions between the candidate components with respect to the primary outcome of interest. The extensive knowledge gained provides an opportunity to remove ineffective components, or adjust component settings, so that the final intervention package has been optimized before it is evaluated in expensive randomized controlled trials.

### 1.9. Objective and aims

**Figure 1:**
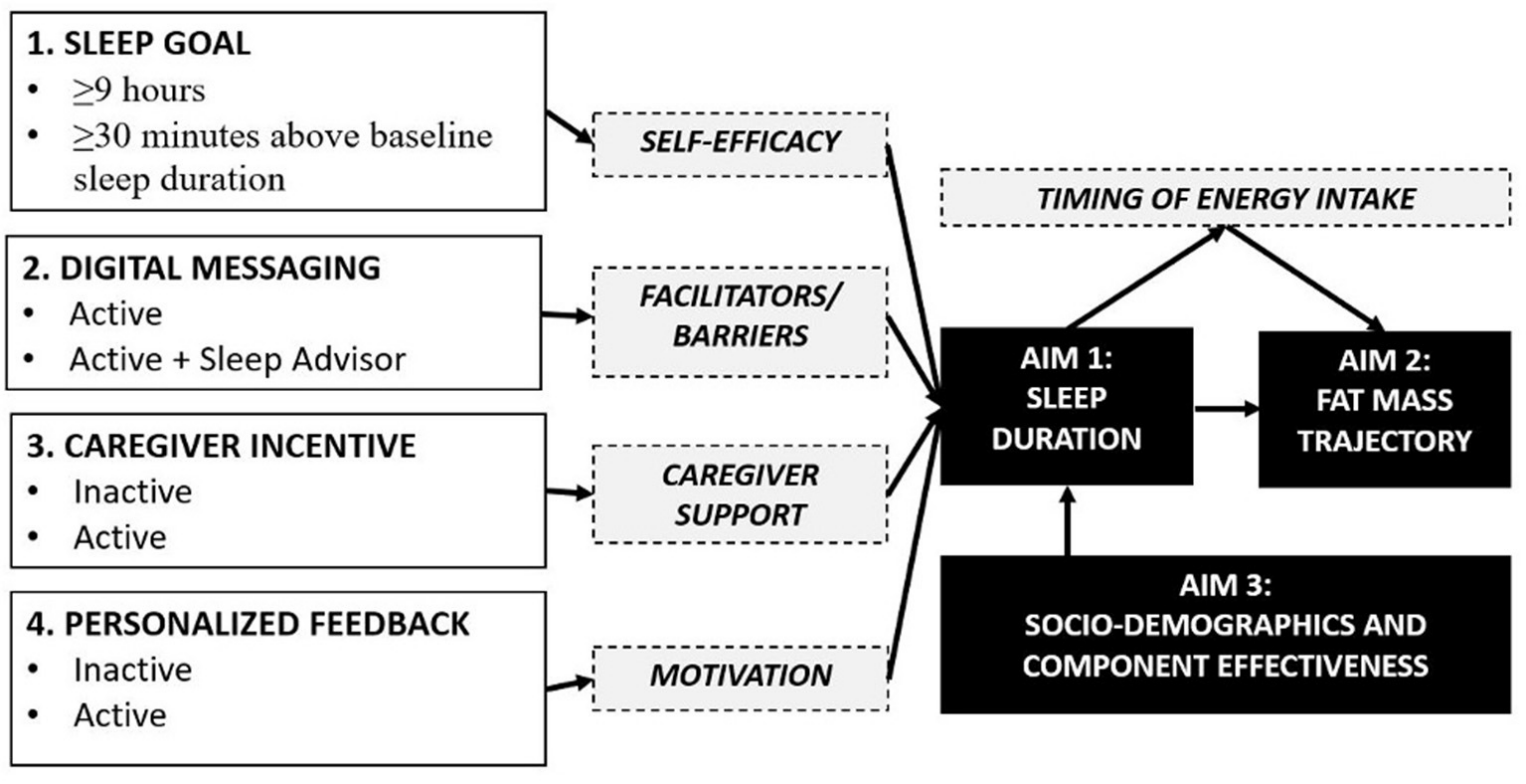
Conceptual model of candidate component settings and mediators for child sleep promotion and obesity prevention.

In this paper we provide the protocol for the Sleep Extension and Enhancement Possibilities for Youth (SLEEPY) 2.0 study. This is an optimization trial designed to determine the optimal component settings for a mobile health platform, for the pediatric care setting, to promote longer sleep duration for childhood obesity prevention. The conceptual model is provided in Figure 1. *Under aim 1*, we will determine the optimal settings for sleep promotion by assessing four candidate intervention components, using a randomized 2^4^ factorial design: (1) a sleep goal component, (2) a digital sleep guidance messaging component, (3) a caregiver-directed incentive component, and (4) a performance feedback component. We hypothesize that we will identify component settings that effectively increase sleep duration. *Under aim 2*, we will determine if the optimal settings for sleep promotion improve fat mass trajectories. Fat mass will be measured at baseline, 6-, and 12-months, and diet intake and behavior will be measured for mechanistic insights. We hypothesize that component settings optimal for sleep will lower gains in fat mass. Finally, *under aim 3*, we will determine if the optimal settings for sleep promotion are equally effective across sociodemographic groups, including the neighborhood context. We hypothesize that component settings that are optimal for sleep will be equitable across sexes, racial/ethnic backgrounds, and neighborhood contexts. Our proposed research will greatly advance the field of behavioral sleep medicine and reimagine how insufficient sleep duration is managed, and obesity is prevented in pediatric healthcare.

## 2. Materials and equipment

### 2.1. Sleep tracker

Participants will be asked to self-monitor their sleep patterns over the entire study period, using an ActiGraph Insight device. The Insight devices are worn on the wrist and use accelerometry to measure arm movements that can infer sleep and wake periods. The data collected on the Insight device will be uploaded into ActiGraph's cloud-based database called CenterPoint using Bluetooth and a smartphone application. The data will be processed in CenterPoint to generate estimates of sleep using the validated Cole-Kripke algorithm for sleep assessment (Cole et al., 1992; Quante et al., 2018). The sleep data will then be transferred to the Way to Health platform using a web API.

### 2.2. Way to health

Way to Health (W2H) is an automated information technology platform that integrates wireless devices, clinical trial randomization, messaging (text and e-mail), and secure data capture for research purposes (Asch and Volpp, 2012). This was designed by faculty and staff at the Leonard David Institute Center for Health Incentives and Behavioral Economics (CHIBE) at the University of Pennsylvania to create an efficient, scalable, and low-cost way to test behavioral interventions using a platform that can be deployed anywhere in the United States. The following features of the W2H Platform will be used in this study: online and mobile participant enrollment; integration with ActiGraph sleep tracker; computerized randomization of participants; automated communication with participants via text; delivery of incentives to encourage behavior change; and online research and electronically secured data.

### 2.3. REDCap

REDCap is a secure, HIPAA-compliant application for data collection and storage that provides logging of all project activity and data entry history for all records, as well as HIPAA-compliant security and daily backups. REDCap will be used for screening potential participants, consenting participants, and delivering surveys to enrolled participants.

## 3. Methods

### 3.1. Study design

A 2^4^-factorial design (i.e., 16 study conditions) will be used to address the study objectives (Table 1). A two-week run-in phase will be used to measure baseline sleep duration, a 6-month intervention phase will be used to measure changes in sleep duration from baseline, and a ≈5.5-month follow-up phase will be used to measure if there are lasting changes in sleep duration from baseline. Clinical measurements (e.g., DXA, 24 h dietary recalls) will be obtained at baseline, 6-months, and 12-months at the Children's Hospital of Philadelphia (CHOP).

**Table 1:** The 2^4^ factorial study design.

	**Candidate components**			
**Condition**	**Sleep goal**	**Digital messaging**	**Caregiver incentive**	**Performance feedback**
1	≥9 h	Active	Inactive	Inactive
2	≥9 h	Active	Inactive	Active
3	≥9 h	Active	Active	Inactive
4	≥9 h	Active	Active	Active
5	≥9 h	Active + SA	Inactive	Inactive
6	≥9 h	Active + SA	Inactive	Active
7	≥9 h	Active + SA	Active	Inactive
8	≥9 h	Active + SA	Active	Active
9	≥30 min above baseline	Active	Inactive	Inactive
10	≥30 min above baseline	Active	Inactive	Active
11	≥30 min above baseline	Active	Active	Inactive
12	≥30 min above baseline	Active	Active	Active
13	≥30 min above baseline	Active + SA	Inactive	Inactive
14	≥30 min above baseline	Active + SA	Inactive	Active
15	≥30 min above baseline	Active + SA	Active	Inactive
16	≥30 min above baseline	Active + SA	Active	Active

SA, Sleep Advisor.

### 3.2. Sample

The sample will include 325 typically developing children and one of their caregivers. To be eligible the children must be aged 8–12 years, have a body mass index (BMI) greater than or equal to the 50^th^ and less than the 95^th^ percentile, and typically sleep <8.5 h per night. Children will be excluded if they have a diagnosis of a major medical condition and/or take medications that impact sleep. Caregivers must be available to put their child to bed on the majority of nights during the study and have smartphone, tablet, or computer access for the duration of the intervention. Table 2 lists the full inclusion and exclusion criteria. Eligibility will be assessed using a screening questionnaire, with the exception for BMI, which will be assessed using measured height and weight at the baseline study visit, and sleep duration, which will be assessed during the run-in phase.

**Table 2:** Inclusion and exclusion criteria for children and caregivers by eligibility assessment phases.

**Screening phase inclusion criteria for children**	**Children aged 8 to 12 years**
	Speak, read, and write in English
	Parental/guardian permission (informed consent) and child assent
Screening phase exclusion criteria for children	A clinically diagnosed chronic disease falling under one or more of the following categories: congenital birth defects, syndromes, or abnormalities; cardiac, hematological, oncological, immunological, pulmonary, renal, rheumatological, or musculoskeletal conditions; endocrine, gastrointestinal, metabolic, sleep, neurodevelopmental, or weight disorders
	Children regularly taking medications related to exclusionary medical conditions or that impact sleep
	Children who will transition to high school during the study
	Children who share a bed
	Children enrolled in another clinical trial
	Have a parent/guardian works, or has a family member who works, for the Division of Gastroenterology, Hepatology and Nutrition at CHOP
Screening phase inclusion criteria for caregivers	Be the parent/guardian of an eligible child enrolled in the main study
	Speak, read, and write in English
	Be available to but the child to bed on the majority of nights
	Have access to a smartphone to install the sleep tracking application for the duration of the study
Baseline study visit inclusion criteria for children	BMI ≥ 50^th^ and <90^th^ percentile for age and sex
Run-in period inclusion criteria for children	Sleep duration ≤ 8.5 hours per night on average

BMI, Body Mass Index; CHOP, Children's Hospital of Philadelphia.

### 3.3. Study timeline

The study will be conducted in 5 phases: screening, run-in, randomization, intervention, and follow-up. The study procedures occurring during each phase are illustrated in Table 3, and the study activities completed during each study phase are described below.

**Table 3:** Study procedures across the 12-month study period.

**Study phase**	**Screening**	**Run-In**		**Rand**.	**Intervention**							**Follow-up**						
**Where**	**Home**	**CHOP visit 1**	**Home**	**Home**	**Home**						**CHOP visit 2**	**Home**						**CHOP Visit 3**
**Study month**	**N/A**	**0**	**0^a^**	**0**	**1**	**2**	**3**	**4**	**5**	**6**	**6**	**7**	**8**	**9**	**10**	**11**	**12**	**12**
Screening questionnaire	X																	
Informed consent/assent		X																
Anthropometry		X^b^									X							X
DXA scan		X^c^									X							X
Child and caregiver questionnaires		X									X							X
Wear sleep tracker			X^d^		X	X	X	X	X	X	X	X	X	X	X	X	X	X
Sleep diary			X			X		X		X			X		X		X	
Dietary recall			X						X							X		
Randomization (if eligible)			X	X														
Intervention engagement					X	X	X	X	X	X								
Monthly surveys					X	X	X	X	X	X	X^e^	X	X	X	X	X	X	X^e^

^a^Data will be collected during the 2nd week of the run-in period. ^b^Confirm BMI eligibility from the baseline anthropometry measures. ^c^Pregnancy tests will be administered to female participants who are 11 years and female participants younger than 11 years who have begun menses. ^d^Confirm insufficient sleep duration eligibility from sleep tracker data collected during the run-in phase. ^e^Monthly survey items will be completed at Visit 2 or Visit 3 if the surveys were not completed at home during months 6 or 12, respectively.

#### 3.3.1. Visit 1

Participants who pass the screening questionnaire will be invited to CHOP for an in-person study visit. Prior to any research procedures, the caregiver and child will complete the informed consent/assent process. As the first procedures, height and weight measures will be taken to determine BMI eligibility. Next, the sitting height and waist circumference measures will be measured, followed by the DXA scan and questionnaires. The caregiver will also complete questionnaires. Before leaving, the caregiver will be provided with a W2H account and the child will be given a sleep tracking device. A smartphone application that is needed to collect the sleep tracker data will be installed on the caregiver's smartphone, tablet, or computer.

#### 3.3.2. Run-in phase

Children will be asked to maintain their usual sleep patterns for a 2-week period to capture their baseline sleep duration. It is expected that sleep duration may increase upon self-monitoring sleep, so the 2nd week of the run-in phase will define each participant's baseline sleep duration. If sleep duration measured by the sleep tracker is greater than or equal to 8.5 h per night on average during the second run-in week they will be excluded. In addition, if <2 nights of data are provided during the second run-in week, they will be excluded due to poor compliance. Otherwise, they will be randomized to a study condition for the optimization trial.

Additionally, baseline dietary intake will be captured during the 2nd week of run-in phase via three 24-h dietary recalls. A self-reported sleep diary will also be completed during the 2nd week of the run-in phase.

#### 3.3.3. Randomization and blinding

A computerized random number generated by the W2H platform will assign children to 1 of 16 study conditions. Block randomization will be used. Participants cannot be blinded to the interventions components to which they are exposed. The study coordinators will not be blinded because they will need to monitor intervention delivery and communicate appropriately with families. Investigators will be blinded, except the psychologist on the team who needs to direct the virtual study visit delivery for one of the intervention components.

#### 3.3.4. Intervention phase

Children will be randomly assigned to 1 of 16 conditions for a 6-month intervention phase. The study conditions differ with respect to sleep goal (guideline-based or personalized), digital sleep guidance (active or active with virtual sleep advisor phone calls), caregiver-directed incentive (inactive or active), and performance feedback (inactive or active). The study design includes one constant: all participants need to self-monitor their sleep duration by wearing the sleep tracker to allow for daily capture of sleep duration and goal achievement status. Dietary intake will be captured during the final month of the intervention phase via three 24-h dietary recalls. Sleep diaries will be completed by all participants for 1 week in months 2, 4, and 6. Participants will also complete monthly surveys at the end of each month.

#### 3.3.5. Visit 2

The caregiver and child will complete questionnaires. In addition, the child will complete anthropometric measures and a DXA scan. These measures will all be the same as those completed at Visit 1. This visit will be scheduled to occur 6-months after randomization, with a 4-week window in anticipation of scheduling issues.

#### 3.3.6. Follow-up phase

Participants will be asked to continue to wear the sleep tracker device to capture sleep patterns with all intervention components inactivated for ≈5.5 months follow-up phase. The purpose is to determine if any changes in sleep duration persist over time.

Dietary intake will be captured during the final month of the follow-up phase via three 24-h dietary recalls. Sleep diaries will be completed by all participants for 1 week in months 8, 10, and 12. Participants will also complete monthly surveys at the end of each month to continue to assess self-reported sleep data (PROMIS surveys) and mediators.

#### 3.3.7. Visit 3

The caregiver and child will complete questionnaires. In addition, the child will complete anthropometric measures and a DXA scan. These measures will all be the same as those completed at Visits 1 and 2. This visit will be scheduled to occur 12-months after randomization, with a 4-week window in anticipation of scheduling issues.

### 3.4. Study procedures and measures

#### 3.4.1. Anthropometry

Trained staff in CHOP's Nutrition and Growth Lab conduct standard body measurements. Height and weight will be measured first. From the height and weight data, a study staff member will verify that the child is eligible to proceed based on their BMI using the CDC's online calculator. If eligible to proceed, the remaining anthropometric measures, sitting height and waist circumference, will be completed.

#### 3.4.2. DXA scan

A DXA scanner will be used to measure the amount of fat, lean and bone tissue in each participant. This method uses a very low powered X-ray beam to scan the entire body. The whole-body scan takes <5 min. In the event of movement during the scan, technicians will attempt to repeat a scan up to two times (a total of 3 tries). The radiation used in this machine is very minimal. This is the standard method for measuring fat mass and body composition and is considered safe for children. Note, prior to having a DXA scan, a urine pregnancy test will be performed for female participants greater than or equal to 11 years of age and female participants younger than 11 years who are physically capable of becoming pregnant.

#### 3.4.3. Dietary recalls

Trained dieticians will call the participants three times during run-in (2^nd^ week), intervention (month 5), and follow-up phases (month 11). The participants will be asked to recall the foods and drinks they have consumed in the past 24 h. This dietary intake data will be entered into Nutrient Data System for Research (NDSR) to derive dietary patterns and estimates of energy, macronutrient, and micronutrient intake at the food, meal (which are time-stamped), day, visit, and participant levels.

#### 3.4.4. Sleep tracker

Sleep trackers will be issued at the baseline visit and will be worn for the duration of the study (approximately 1 year). Participants will be able to view some of their sleep tracker data on their W2H dashboard. All participants will see their sleep duration and the number of times they have met their sleep goal. Those in the performance feedback condition will also see more in-depth performance metrics (e.g., percentage of sleep goal achieved).

#### 3.4.5. Sleep diary

Participants will be asked to complete sleep diaries over 7 days during run-in (2^nd^ week), intervention (months 2, 4, and 6) and follow-up phases (months 8, 10, and 12). The sleep diary includes a “morning page” with 12 items to capture self-reported sleep health metrics (e.g., how well they slept last night) and an “evening page” with 5 items asking about alertness, nap times, and caffeine consumption during the day. The sleep diary will be sent via text message through REDCap using an encrypted, participant specific link. Twilio Cloud Communications will be used for texting.

#### 3.4.6. Child questionnaires

Research staff will assist children with the completion of their questionnaires at the in-person visits at baseline, 6-months, and 12-months. These questionnaires are broken into 8 sections: Sociodemographics and Home Environment; Bedroom Environment; Sleep and Chronotype; Screen Time and Social Media; Eating Behavior; School and Homework; Physical Activity; and Depression and Anxiety. The specific instruments included in the child questionnaires are listed in Table 4.

**Table 4:** Instruments included in the child and caregiver questionnaires.

**Child questionnaires**	**Caregiver questionnaires**
Eating in the absence of hunger questionnaire (Tanofsky-Kraff et al., 2008)	Confusion, hubbub, and order scale (Chatterjee et al., 2015)
Evaluation of activity survey in youth (Pate et al., 2018)	Eating in the absence of hunger questionnaire (Tanofsky-Kraff et al., 2008)
Munich chronotype questionnaire (Zavada et al., 2005)	Epworth sleepiness scale (Johns, 1991, 1992)
PROMIS pediatric anxiety short form 8a (Irwin et al., 2010)	International physical activity questionnaire (Craig et al., 2003; Lee et al., 2011)
PROMIS pediatric depressive symptoms short form 8a (Irwin et al., 2010)	Morning-eveningness questionnaire (Horne and Östberg, 1976)
PROMIS pediatric sleep disturbance short form 8a (Forrest et al., 2018)	Physical activity neighborhood environment scale (Sallis et al., 2010; Ding et al., 2013)
PROMIS pediatric sleep-related impairment short form 8a (Forrest et al., 2018)	
School sleep habits survey (Wolfson et al., 2003)	
The hunger vital sign, U.S. household food security survey (Hager et al., 2010)	
Youth risk behavior survey (Control and Prevention, 2016; Mpofu et al., 2023)	

#### 3.4.7. Caregiver questionnaires

Caregivers will complete questionnaires during each in-person visit at baseline, 6-months, and 12-months. These questionnaires are broken into 7 sections: Sociodemographics; Pregnancy, Birth, and Infancy; Home Environment; Neighborhood Environment; Eating Behavior; Physical Activity; and Sleep and Health. The specific instruments included in the caregiver questionnaire are listed in Table 4.

#### 3.4.8. Monthly questionnaires

At the end of every month during the intervention and follow-up phases, caregivers will receive a text message with a link for 3 surveys in REDCap. These questionnaires include the Monthly Mediator Survey, Notable Events Form, and PROMIS sleep measures. If the participant did not complete any of these surveys the month leading up to the 6-month and 12-month in-person study visits, they will be administered at the in-person visit.

#### 3.4.9. Geospatial measures

Each participant's primary home address will be geocoded using ArcMap (v.10.5.1). To link built environment exposures, we will define each participants' residential neighborhood as: (1) the census block group in which their primary home is located, and (2) a half-mile buffer around their home address based on Euclidean (straight-line) distances. These definitions are being used because we expect that the built environment features influencing sleep will operate at close proximity. We will consider alternative buffers/neighborhood definitions in sensitivity analyses. The following geospatial measure will be calculated, but it is noted that these may be subject to change based on updated reference data sets and new data sets that become available.

##### 3.4.9.1. Sound levels

Sound data will be obtained from the US National Park Service Sound Map.^1^ The Park Service used random forest models to predict expected sound levels across the contiguous US on a typical summer day with calm conditions based on empirical acoustical data from 479 locations in national parks, urban and suburban areas. Their model incorporated geospatial features and was validated using leave-one-out cross validation (Mennitt and Fristrup, 2016). The specific sound metric used was L50 sound pressure level, which reflects the median sound level across all summer daytime seconds recorded for a given location after adjustment for the sensitivity of human hearing to very low and high frequencies (“A-weighting”).^2^ We will calculate neighborhood-level sound levels by taking the average sound levels in A-weighted decibels within a half-mile buffer of each participants' residence.

##### 3.4.9.2. Tree canopy cover

Data on tree canopy cover will be obtained from 2016 National Land Cover Database that was create by the US Forest Service.^3^ A raster file reflecting the estimated percentage tree canopy coverage at a 30-m resolution for the continuous US estimates were produced using random forest regression models incorporating multi-spectral Landsat satellite imagery and elevation data (Coulston et al., 2012). We will calculate the average percentage tree canopy cover within a half-mile buffer of each participants' home.

##### 3.4.9.3. Street connectivity

Street connectivity will be assessed by street density and intersection density using data obtained using Esri's 2016 StreetMap Premium (Esri, Redlands, CA). Street and intersection density will be calculated by dividing (1) the total linear meters of road in the half-mile buffer and (2) the total number of intersections in the buffer, by the buffer area in hectares.

##### 3.4.9.4. Population and housing density

Block group-level population density and housing density will be calculated using data from the 2012–2016 American Community Survey by dividing each participants' census block group population and number of housing units, respectively, by its land area in square miles.

##### 3.4.9.5. Neighborhood poverty

The percentage of residents in participants' home census tract with household incomes below the federal poverty limit will be calculated using the US Census Bureau's 2012–2016 American Community Survey data. These data will be retrieved from the IPUMS National Historical Geographic Information System (Manson et al., 2019).

#### 3.4.10. Candidate intervention components

The conceptual model showing each candidate component and their mediator is provided in Figure 1. For the sleep goal component, participants will be randomized to a condition that has either a guideline-based goal (≥9 h per night) or a personalized goal (+30 min per night above baseline). The sleep goal is specific to weeknights and the total number of times the sleep goal is achieved for each participant will be calculated.

For the digital sleep guidance component, participants will be randomized to receive text messages only or text messages with virtual sleep advisor phone calls. The text messages focus on evidence-based sleep health recommendations and focus span 6 domains: age-appropriate bedtime and consistency, schedules and routines, location, electronics, exercise and diet, and positivity and relaxation (Allen et al., 2016; Meltzer et al., 2021b). The sleep advisor phone calls will consist of three telephone calls of ≈15–30 min each during months 1, 2, and 5 of the intervention period. This brief intervention approach is modeled on previous sleep health education research (Quach et al., 2011; Corkum et al., 2016; Mindell et al., 2016). The calls in months 1 and 2 will be used to reinforce sleep health education and message content and to provide personalized guidance and problem-solving around overcoming barriers to increased sleep duration. The last call in month 5 will focus on how to sustain sleep health habits into the future. The sleep health sessions will narrow in on 1–2 barriers that are personally preventing a child from sleeping sufficiently.

For the caregiver-directed incentive component, participants will be randomized to receive or not receive a loss-framed financial incentive. If activated, caregivers will receive a financial incentive when their child achieves their sleep goal. The incentive will be loss-framed; caregivers will receive an endowment of $10 at the start of each intervention week, in a virtual bank account; we will deduct $2 each weeknight the sleep duration goal is not met; the funds remaining in the virtual account will be dispensed each Sunday. The weekly endowment-payment approach allows for fresh starts each week. The incentive will be directed at caregivers as a method to enhance engagement in helping their child to increase their sleep duration.

For the performance feedback component, participants will be randomized to receive or not receive detailed performance feedback metrics. All participants will be informed of their sleep duration. Those randomized to enhanced performance feedback will be able to view additional metrics on their W2H Dashboard and via text message, including sleep timing data, percentage of sleep goal achieved, shortest night of sleep, longest night of sleep. They will also be able to earn badges during the intervention in recognition of their progress. Badges will be sent via text message for (1) notable increases in sleep duration (i.e., bronze, silver, or gold medal badges for 1–15 min, 16–30 min, and >30 min average increases in sleep duration in the past week); (2) achieving their sleep goal for the first time; and (3) in recognition of the number of times they achieved their sleep goal in the past week.

### 3.5. Outcomes

#### 3.5.1. Primary outcomes

The primary outcomes are nighttime sleep duration (hours per night) on weeknights and fat mass index (kg/m^2^). Sleep duration will be estimated using the sleep tracker. Up to 260 weeknights (Sun-Thurs) and 104 weekend nights (Fri and Sat) of data will be captured per participant. Federal holidays and notable events (e.g., vacations) occurring on weeknights during the school term will be re-coded as weekend nights. Weeknights are the focus since insufficient sleep among children is more prevalent on weeknights.

Total body fat mass will be measured by DXA (Hologic Discovery A; Hologic Inc., Bedford, MA, operating in software version 13.5.3) at baseline, 6-months, and 12-months. Fat mass index (FMI, kg of fat divided by height in meters squared) will be calculated, and age and gender specific FMI Z-scores will be determined using U.S. specific FMI growth charts (Weber et al., 2013).

##### 3.5.1.1. Optimization objective

The optimal component settings will have to meet the following criteria: increase average baseline sleep duration on weeknights by ≥30 min for ≥75% of intervention weeks and for ≥50% of follow-up weeks. A 30-min increase in sleep duration has been shown to be feasible in our prior research (Mitchell et al., 2021) and has been shown to improve alertness and emotional regulation (Gruber et al., 2012) and to lower weight gain (Hart et al., 2022).

#### 3.5.2. Secondary outcomes

Secondary outcomes include sleep timing metrics from the sleep tracker to understand if any changes in sleep timing lead to changes in sleep duration. The sleep timing metrics include sleep onset on weeknights and weekend nights (hours from 00:00), sleep offset on weeknights and weekend nights (hours from 00:00), and sleep midpoint on weeknights and weekend nights (hours from 00:00). “Social jetlag” will be calculated by subtracting the weekend night sleep midpoint from the weeknight sleep midpoint. Social jetlag provides an indication of the discrepancy between internal biological clocks and social requirements and has been linked to cardiometabolic risk (Cespedes Feliciano et al., 2019; Mathew et al., 2020). Sleep quality metrics will also be captured from the sleep tracker on weeknights and weekend nights: sleep efficiency (percentage of time spent asleep during overnight sleep periods), sleep onset latency (time to fall asleep), and wake after sleep onset (time awake during overnight sleep periods).

Secondary outcomes also include sleep disturbance and sleep impairment measured using the Patient-Reported Outcomes Measurement Information System (PROMIS) item bank for pediatric sleep (Forrest et al., 2018). This validated survey generates a sleep disturbance T-score based on items related to sleep onset, sleep continuity and sleep quality in the past week. The survey also generates a sleep-related impairment T-score based on items related to daytime sleepiness, cognition, affect and behavior, and daytime activities in the past week. Higher T-scores indicates poorer sleep quality (Forrest et al., 2018). Participants will complete this survey at baseline and at the end of each month during the intervention and follow-up phases.

Secondary outcomes will also be considered with respect to body composition and size. These include visceral body adipose tissue area (cm^2^), waist circumferences (cm), and BMI (kg/m^2^).

### 3.6. Power calculation

#### 3.6.1. Aim 1

The primary outcome for Aim 1 is nighttime sleep duration (hours per night) on weeknights (measured daily by the sleep tracker). The “MOST” R package (FactorialPowerPlan) was used to determine the sample size needed to detect a 30 min per night difference in nighttime sleep duration on weeknights (alpha = 0.05; power = 0.8; pretest-posttest correlation of sleep duration = 0.7; and a standard deviation sleep duration = 0.7 h per night). The effect size and parameters were derived from our prior studies. The calculation accounted for detection of main effects plus all possible interactions. The power calculation revealed that a minimum sample size of 41 was needed for Aim 1.

#### 3.6.2. Aim 2

The primary outcome for Aim 2 is fat mass index (kg/m^2^) (measured at baseline, 6-months, and 12-months by DXA). The “MOST” R package (FactorialPowerPlan) was used to determine the sample size needed to detect a 0.25 kg/m^2^ reduction in fat mass index at 6-months and 12-months (alpha = 0.05; power = 0.8; pretest-posttest correlation of fat mass index = 0.9; and a standard deviation for fat mass index = 0.9). The effect size and parameters were derived from our prior studies. The calculation accounted for detection of main effects plus all possible interactions. The power calculation revealed that a minimum sample size of 282 was needed for Aim 2.

#### 3.6.3. Aim 3

The primary outcome for Aim 3 is nighttime sleep duration (hours per night) on weeknights (measured daily by the sleep tracker). It has been observed that male children sleep on average 12 min per night less than female children, and that African American/Black children sleep on average 33 min per night less than non-Latinx White children (Guglielmo et al., 2018; Billings et al., 2021; Min et al., 2021). We will determine if the effectiveness of the optimal component settings for sleep promotion differ by ≥20 min across sociodemographic factors. This calculation revealed that 286 participants are needed to detect a ≥20 min per night difference in the effectiveness of the optimal component settings for sleep duration by higher and lower sociodemographic levels. The calculation was completed in Stata (version 14.2) using the “power repeated” command to compute the sample size. The parameters used for the calculation were: alpha = 0.05, power = 0.8, number of repeated measures = 52, number of groups = 2, error variance = 1.1, and correlation = 0.7.

#### 3.6.4. Final sample size

We elected to randomize 325 children. Accounting for an attrition rate of 10%, the study has a sample size that is powered to detect clinically relevant differences in the primary outcomes under each aim.

### 3.7. Statistical plan

Under the intent to treat principle, we will use all available data and participants under the assumption of missingness at random. No participants will be excluded from the analysis except for the unlikely case of no outcome measurements for a participant. Final models will be adjusted for covariate dependent missingness due to dropout. We will examine sensitivity to the assumptions about the missing data mechanism.

#### 3.7.1. Aim 1

Mixed-effects linear regression models will be used to determine if the intervention components are associated with changes in weeknight sleep duration from baseline. The fixed model components will include indicators for each factor (with effect coding used to identify each component level), time in weeks, and all possible interactions between time and factors. The full model will be reduced to a more parsimonious model by selecting the main effects and interactions achieving a Type I error rate of alpha = 0.05.

#### 3.7.2. Aim 2

Mixed-effects linear regression models will be used to measures changes in fat mass index using the most parsimonious model from Aim 1 (i.e., optimal component settings for sleep promotion).

#### 3.7.3. Aim 3

The most parsimonious model from Aim 1 (i.e., optimal component settings for sleep promotion), will be expanded to determine if effectiveness differs by sociodemographic factors. The fixed model components will include indicators for each component (effect coding to represent the levels of each factor), time in weeks, the sociodemographic factor of interest, interactions between time, component(s), and sociodemographic factor. The sociodemographic factors that will be modeled include sex, race/ethnicity, and neighborhood context (poverty, sound, canopy cover, housing and population density, and street connectivity).

## 4. Anticipated results

Expected outcomes include identifying optimal component settings for sleep promotion in children. This increase will be clinically meaningful with improvements in fat mass trajectories detected. Importantly, the platform will have broad impact by promoting sleep health equity across sociodemographic groups (Jackson et al., 2020). With the optimal settings identified, we will be able to determine the effectiveness of the final intervention package under the evaluation phase of the MOST framework in a future trial.

## 5. Discussion

Our long-term goal is to develop, evaluate and continually improve pediatric sleep extension interventions that are scalable and can be widely disseminated in pediatric primary care. Upon completion of this study, we anticipate that we will have optimized the first behavioral sleep extension package for pediatric primary care to help prevent childhood obesity. A potential immediate next step will be to complete the evaluation phase of the MOST framework, where we will conduct an RCT to determine if our optimized intervention package causes improvements in sleep duration and lower gains in fat mass over time, equitably across sociodemographic groups. We will also remain committed to the MOST framework and the related continuous optimization principle. All behavioral interventions can be improved, and we will seek to complete another cycle of optimization for equitable sleep promotion in other obesity related disease areas.

## Data availability statement

The original contributions presented in the study are included in the article/supplementary material, further inquiries can be directed to the corresponding author.

## Author contributions

MF: Project administration, Visualization, Writing—original draft. JD: Project administration, Visualization, Writing—review and editing. AF: Conceptualization, Methodology, Writing—review and editing. SM: Conceptualization, Methodology, Writing—review and editing. KM: Data curation, Methodology, Writing—review and editing. AW: Conceptualization, Writing—review and editing. JM: Conceptualization, Funding acquisition, Investigation, Methodology, Project administration, Resources, Supervision, Writing—original draft.

## Footnotes

^1^U.S. National Park Service. *Mapping Sound - Natural Sounds*. U.S. National Parks Service. Available online at: https://www.nps.gov/subjects/sound/soundmap.htm (accessed January 13 2020).

^2^National Park Service. *Mapping Sounds: Frequently Asked Questions*. National Park Service. Available online at: https://www.nps.gov/subjects/sound/mapfaq.htm (accessed March 3 2020).

^3^Multi-Resolution Land Characteristics Consortium. *Nlcd 2016 Usfs Tree Canopy Cover (Conus)* [Online]. Multi-Resolution Land Characteristics Consortium. Available online at: https://www.mrlc.gov/data/nlcd-2016-usfs-tree-canopy-cover-conus (accessed January 13, 2020).
